# North American import? Charting the origins of an enigmatic *Trypanosoma cruzi* domestic genotype

**DOI:** 10.1186/1756-3305-5-226

**Published:** 2012-10-10

**Authors:** Federico A Zumaya-Estrada, Louisa A Messenger, Teresa Lopez-Ordonez, Michael D Lewis, Carlos A Flores-Lopez, Alejandro J Martínez-Ibarra, Pamela M Pennington, Celia Cordon-Rosales, Hernan V Carrasco, Maikel Segovia, Michael A Miles, Martin S Llewellyn

**Affiliations:** 1 Centro Regional de Investigación en Salud Pública, Instituto Nacional de Salud Pública, Tapachula, Chiapas, México; 2 London School of Hygiene and Tropical Medicine, London, UK; 3 Department of Biology, University of Maryland, College Park, MD, USA; 4 Área de Entomología Médica, Centro Universitario del Sur, Universidad de Guadalajara, Ciudad Guzmán, Jalisco, México; 5 Center for Health Studies, Research Institute, Universidad del Valle de Guatemala, Guatemala City, Guatemala; 6 Instituto de Medicina Tropical, Universidad Central de Venezuela, Caracas, Venezuela

**Keywords:** Trypanosoma cruzi, Maxicircle, Microsatellite, Chagas Disease, Phylogeography, Population genetics, TcI

## Abstract

**Background:**

*Trypanosoma cruzi*, the agent of Chagas disease, is currently recognized as a complex of six lineages or Discrete Typing Units (DTU): TcI-TcVI. Recent studies have identified a divergent group within TcI - TcI_DOM_. TcI_DOM._ is associated with a significant proportion of human TcI infections in South America, largely absent from local wild mammals and vectors, yet closely related to sylvatic strains in North/Central America. Our aim was to examine hypotheses describing the origin of the TcI_DOM_ genotype. We propose two possible scenarios: an emergence of TcI_DOM_ in northern South America as a sister group of North American strain progenitors and dispersal among domestic transmission cycles, or an origin in North America, prior to dispersal back into South American domestic cycles. To provide further insight we undertook high resolution nuclear and mitochondrial genotyping of multiple Central American strains (from areas of México and Guatemala) and included them in an analysis with other published data.

**Findings:**

Mitochondrial sequence and nuclear microsatellite data revealed a cline in genetic diversity across isolates grouped into three populations: South America, North/Central America and TcI_DOM_. As such, greatest diversity was observed in South America (A_r_ = 4.851, π = 0.00712) and lowest in TcI_DOM_ (A_r_ = 1.813, π = 0.00071). Nuclear genetic clustering (genetic distance based) analyses suggest that TcI_DOM_ is nested within the North/Central American clade.

**Conclusions:**

Declining genetic diversity across the populations, and corresponding hierarchical clustering suggest that emergence of this important human genotype most likely occurred in North/Central America before moving southwards. These data are consistent with early patterns of human dispersal into South America.

## Findings

*Trypanosoma cruzi*, the aetiological agent of Chagas disease, infects 6-8 million people in Latin America, while some 25 million more are at risk of acquiring the disease [1]. Parasite transmission to mammal hosts, including humans, can occur through contact with the faeces of hematophagous triatomine bugs. However, non-vectorial routes are also recognized, including blood transfusion, organ transplantation, congenital transmission, and oral transmission via ingestion of meals contaminated with infected triatomine feces [2,3].

*T. cruzi* (family Trypanosomatidae; Euglenozoa: Kinetoplastida) is most closely related to several widely dispersed species of bat trypanosomes [4]. Salivarian trypanosomes including medically important *Trypanosoma brucei* subspecies, represent a more divergent group [5]. The age of the split between the *T. cruzi*-containing and *T. brucei*-containing trypanosome lineages is thought to have been concurrent with the separation of Africa and South America/Antarctica/Australasia 100MYA [6], implying that *T. cruzi* and the other Schizotrypanum species evolved exclusively in South America. Others propose an alternative origin of *T. cruzi* from an ancestral bat trypanosome potentially capable of long range dispersal [7]. Whilst the precise scenario for the arrival of ancestral *Schizotrypanum* lineages in South America is a matter for debate, the current continental distribution and genetic diversity of *T. cruzi* supports an origin within South America. Parasite transmission is maintained via hundreds of mammal and triatomine species in different biomes throughout South and Central America, as well as the southern states of the USA [8].

Biochemical and molecular markers support the existence of six lineages or Discrete Typing Units (DTU): TcI, - TcVI agreed by international consensus ( [9]. Each DTU can be loosely associated with a particular ecological and/or geographical framework [10]. TcI is ubiquitous among arboreal sylvatic foci throughout the geographic distribution of *T. cruzi* and is the major agent of human Chagas disease in northern South America. Several molecular tools now identify substantial genetic diversity within TcI [11-14]. Importantly these new approaches consistently reveal the presence of a genetically divergent and homogeneous TcI group (henceforth TcI_DOM_ – previously TcIa/*VEN*_DOM_) associated with human infections from Venezuela to Northern Argentina, and largely absent from wild mammals and vectors sampled to date [14]. The origin of this clade is unclear, although recent work supports a sister group relationship with TcI circulating in North America (e.g. [12,13]).

In this manuscript we have set out to evaluate the genetic diversity of TcI in North/Central America, undertaking a comparison with TcI diversity in South America, including TcI_DOM_. Our aim was to examine hypotheses describing the origin of the TcI_DOM_ clade. We propose two possible scenarios: an emergence of TcI_DOM_ in northern South America as a sister group of North American strains and dispersal among domestic transmission cycles, or an origin in North America, prior to dispersal back into South American domestic cycles, possibly anthropically. To provide further insight into this question we undertook high resolution nuclear and mitochondrial genotyping of multiple Central American strains (from areas of México and Guatemala) and included them in an analysis with other published data [11-13].

A panel of 72 TcI isolates and clones was assembled for analysis (Table 1) [11-16]. Of these, existing sequences and microsatellite data were available for 46 isolates [11,12]. Isolates were classified into three populations: TcI_NORTH-CENT_, TcI_SOUTH_ and TcI_DOM_. TcI_NORTH-CENT_ includes samples from the USA, México, Guatemala and Honduras; TcI_SOUTH_ corresponds to South America (Argentina, Bolivia, Colombia, Venezuela and Brazil) and TcI_DOM_ with exclusively domestic isolates from Colombia and Venezuela, already known to correspond to a genotype with restricted genetic diversity: TcIa, as previously described by Herrera *et al.,* (2007) [17] and *VEN*_Dom_, as described by Llewellyn *et al.,* (2009) [13]. Additional DTU isolates (TcIII-TcIV) were included as out-groups in the mitochondrial analysis.

**Table 1:** ***Trypanosoma cruzi *I samples included in this study**

**Strain code**	**Strain**	**Host/vector**	**Country**	**State**	**Latitude**	**Longitude**	**Date**	**Population**	**Reference**
PALDA4	PALDA4	*Didelphis albiventris*	Argentina	Chaco	-27.133	-61.460	2001	SOUTH	Messenger *et al.*, [12]
PALDA21	PALDA21	*Didelphis albiventris*	Argentina	Chaco	-27.133	-61.460	2001	SOUTH	Messenger *et al.*, [12]
PALDA5	PALDA5	*Didelphis albiventris*	Argentina	Chaco	-27.133	-61.460	2001	SOUTH	Messenger *et al.*, [12]
PALDAV2	PALDAV2^3	*Triatoma infestans*	Argentina	Chaco	-27.133	-61.460	2001	SOUTH	Messenger *et al.*, [12]
PALDA20	PALDA20	*Didelphis albiventris*	Argentina	Chaco	-27.133	-61.460	2001	SOUTH	Messenger *et al.*, [12]
COTMA38	COTMA38	*Akodon boliviensis*	Bolivia	Cotopachi	-17.430	-66.270	2004	SOUTH	Messenger *et al.*, [12]
P234	P234	*Homo sapiens*	Bolivia	Cochabamba	-17.380	-66.160	1985	SOUTH	Messenger *et al.*, [12]
P238	P238	*Homo sapiens*	Bolivia	Cochabamba	-17.380	-66.160	1985	SOUTH	Messenger *et al.*, [12]
P268	P268	*Homo sapiens*	Bolivia	Cochabamba	-17.380	-66.160	1987	SOUTH	Messenger *et al.*, [12]
SJM22	SJM22 cl1	*Didelphis marsupialis*	Bolivia	Beni	-14.810	-64.600	2004	SOUTH	Messenger *et al.*, [12]
SJM34	SJM34	*Didelphis marsupialis*	Bolivia	Beni	-14.810	-64.600	2004	SOUTH	Messenger *et al.*, [12]
SJM37	SJM37	*Didelphis marsupialis*	Bolivia	Beni	-14.810	-64.600	2004	SOUTH	Messenger *et al.*, [12]
SJM39	SJM39 cl3	*Didelphis marsupialis*	Bolivia	Beni	-14.810	-64.600	2004	SOUTH	Messenger *et al.*, [12]
SJM41	SJM41	*Philander opossum*	Bolivia	Beni	-14.810	-64.600	2004	SOUTH	Messenger *et al.*, [12]
SJMC12	SJMC12	*Philander opossum*	Bolivia	Beni	-14.810	-64.600	2004	SOUTH	Messenger *et al.*, [12]
XE5167	XE5167 cl1	*Didelphis marsupialis*	Brasil	Para	-1.710	-48.880	1999	SOUTH	Messenger *et al.*, [12]
IM4810	IM4810	*Didelphis marsupialis*	Brasil	Manaus	-3.070	-60.160	2002	SOUTH	Messenger *et al.*, [12]
B2085	B2085	*Didelphis marsupialis*	Brasil	Belem	-1.360	-48.360	1991	SOUTH	Messenger *et al.*, [12]
XE2929	XE2929	*Didelphis marsupialis*	Brasil	Pará	-5.830	-48.030	1988	SOUTH	Messenger *et al.*, [12]
AAA1cl5	AAA1cl5	*Rhodnius prolixus*	Colombia	Casanare	4.150	-71.200	2010	SOUTH	Ramirez *et al.,* Molecular Ecology *In press*
AAA7cl2	AAA7cl2	*Rhodnius prolixus*	Colombia	Casanare	5.100	-71.600	2010	SOUTH	Ramirez *et al.,* Molecular Ecology *In press*
AAB3cl3	AAB3cl3	*Rhodnius prolixus*	Colombia	Casanare	4.150	-71.200	2010	SOUTH	Ramirez *et al.,* Molecular Ecology *In press*
AAC1cl3	AAC1cl3	*Rhodnius prolixus*	Colombia	Casanare	5.100	-71.600	2010	SOUTH	Ramirez *et al.,* Molecular Ecology *In press*
AACf1cl4	AACf1cl4	*Canis familiaris*	Colombia	Casanare	5.100	-71.600	2010	SOUTH	Ramirez *et al.,* Molecular Ecology *In press*
AAD6cl6	AAD6cl6	*Rhodnius prolixus*	Colombia	Casanare	5.100	-71.600	2010	SOUTH	Ramirez *et al.,* Molecular Ecology *In press*
CACQcl7	CACQcl7	*Homo sapiens*	Colombia	Santander	6.963	-73.420	2009	TcIDOM	Ramirez *et al.,* Molecular Ecology *In press*
CACQcl8	CACQcl8	*Homo sapiens*	Colombia	Santander	6.644	-73.654	2009	TcIDOM	Ramirez *et al.,* Molecular Ecology *In press*
DYRcl16	DYRcl16	*Homo sapiens*	Colombia	Boyacá	5.640	-72.899	2007	TcIDOM	Ramirez *et al.,* Molecular Ecology *In press*
EBcl11	EBcl11	*Homo sapiens*	Colombia	Boyacá	5.130	-73.119	2007	TcIDOM	Ramirez *et al.,* Molecular Ecology *In press*
FECcl10	FECcl10	*Homo sapiens*	Colombia	Boyacá	5.920	-73.500	2001	TcIDOM	Ramirez *et al.,* Molecular Ecology *In press*
Td3cl11	Td3cl11	*Triatoma dimidiata*	Colombia	Boyacá	6.270	-71.200	2000	TcIDOM	Ramirez *et al.,* Molecular Ecology *In press*
X-1084cl10	X-1084cl10	*Rhodnius prolixus*	Colombia	Boyacá	4.960	-73.630	2010	SOUTH	Ramirez *et al.,* Molecular Ecology *In press*
X-236cl9	X-236cl9	*Rhodnius prolixus*	Colombia	Boyacá	4.960	-73.630	2010	SOUTH	Ramirez *et al.,* Molecular Ecology *In press*
YAS1cl3	YAS1cl3	*Alouatta spp*	Colombia	Casanare	5.300	-72.400	2010	SOUTH	Ramirez *et al.,* Molecular Ecology *In press*
38	38	*Triatoma dimidiata*	Guatemala	Jutiapa	14.287	-89.844	2000	NORTH-CENT	This study
46	46	*Triatoma dimidiata*	Guatemala	Santa Rosa	14.177	-90.303	2001	NORTH-CENT	This study
66	66	*Triatoma dimidiata*	Guatemala	Jalapa	14.633	-89.989	2001	NORTH-CENT	This study
67	67	*Triatoma dimidiata*	Guatemala	Jutiapa	14.287	-89.844	2001	NORTH-CENT	This study
70	70	*Triatoma dimidiata*	Guatemala	Jutiapa	14.287	-89.844	2001	NORTH-CENT	This study
71	71	*Triatoma dimidiata*	Guatemala	Jalapa	14.633	-89.989	2001	NORTH-CENT	This study
83	83	*Triatoma dimidiata*	Guatemala	Chiquimula	14.768	-89.458	2002	NORTH-CENT	This study
95	95	*Triatoma dimidiata*	Guatemala	Chiquimula	14.768	-89.458	2002	NORTH-CENT	This study
100	100	*Triatoma dimidiata*	Guatemala	Santa Rosa	14.177	-90.303	2002	NORTH-CENT	This study
113	113	*Triatoma dimidiata*	Guatemala	Chiquimula	14.768	-89.458	2002	NORTH-CENT	This study
116	116	*Triatoma dimidiata*	Guatemala	Baja Verapaz	15.079	-90.413	2002	NORTH-CENT	This study
154	154	*Triatoma dimidiata*	Guatemala	Alta Verapaz	15.594	-90.149	2002	NORTH-CENT	This study
DAVIScl1	DAVIS 9.90 cl1	*Triatoma dimidiata*	Honduras	Tegucigalpa	14.080	-87.200	1983	NORTH-CENT	Messenger *et al.,* 2012
ANITA II	ANITA	*Triatoma dimidiata*	Mexico	Campeche	19.188	-90.300	2011	NORTH-CENT	This study
CAM6	CAM6	*Triatoma dimidiata*	Mexico	Campeche	19.188	-90.300	2011	NORTH-CENT	This study
CRISTY	CRISTY	*Homo sapiens*	Mexico	San Luis Potosí	22.159	-100.990	2007	NORTH-CENT	This study
MICH1	MICH	*Triatoma dimidiata*	Mexico	Michoacan	19.567	-101.707	2011	NORTH-CENT	This study
NINOA	NINOA	*Homo sapiens*	Mexico	Oaxaca	17.054	-96.714	1994	NORTH-CENT	This study
PLI	PL	*Dipetalogaster maxima*	Mexico	Baja California Sur	26.044	-111.666	2001	NORTH-CENT	This study
QROI	QRO	*Triatoma barberi*	Mexico	Queretaro	20.594	-100.393	1986	NORTH-CENT	This study
TQI	TQ	*Triatoma pallidipennis*	Mexico	Morelos	18.953	-99.223	1991	NORTH-CENT	This study
XAL1	XAL	*Triatoma dimidiata*	Mexico	Veracruz	19.173	-96.133	2003	NORTH-CENT	This study
9209802P	9209802P cl1	*Didelphis marsupialis*	USA	Georgia	32.430	-83.310	1992	NORTH-CENT	Messenger *et al.,* [12]
9307	93070103P cl1	*Didelphis marsupialis*	USA	Georgia	32.430	-83.310	1993	NORTH-CENT	Messenger *et al.,* [12]
ARMA	USAARMA cl3	*Dasypus novemcinctus*	USA	Lousiana	30.500	-91.000	*Unknown*	NORTH-CENT	Messenger *et al.,* [12]
USA	USAOPOSSUM cl2	*Didelphis marsupialis*	USA	Lousiana	30.500	-91.000	*Unknown*	NORTH-CENT	Messenger *et al.,* [12]
9354	9354	*Homo sapiens*	Venezuela	Sucre	10.460	-63.610	1999	TcIDOM	Messenger *et al.,* [12]
11541	11541	*Homo sapiens*	Venezuela	Merida	8.590	-71.230	2003	TcIDOM	Messenger *et al.,* [12]
11713	11713	*Homo sapiens*	Venezuela	Lara	10.233	-69.866	2003	TcIDOM	Messenger *et al.,* [12]
11804	11804	*Homo sapiens*	Venezuela	Portuguesa	9.084	-69.103	2003	TcIDOM	Messenger *et al.,* [12]
10462P2C3	10462P2C3	*Homo sapiens*	Venezuela	Miranda	10.266	-66.485	*Unknown*	TcIDOM	This study
10462P2C7	10462P2C7	*Homo sapiens*	Venezuela	Miranda	10.080	-66.449	*Unknown*	TcIDOM	This study
10968P1C1	10968P1C1	*Homo sapiens*	Venezuela	Sucre	10.406	-63.298	*Unknown*	TcIDOM	This study
ANT3P1C6	ANT3P1C6	*Homo sapiens (oral)*	Venezuela	DC	10.500	-66.951	*Unknown*	SOUTH	This study
M13	M13	*Didelphis marsupialis*	Venezuela	Barinas	7.500	-71.230	2004	SOUTH	Messenger *et al.,* [12]
M16	M16 cl4	*Didelphis marsupialis*	Venezuela	Barinas	7.500	-71.230	2004	SOUTH	Messenger *et al.,* [12]
M18	M18	*Didelphis marsupialis*	Venezuela	Barinas	7.500	-71.230	2004	SOUTH	Messenger *et al.,* [12]
M7	M7	*Didelphis marsupialis*	Venezuela	Barinas	7.500	-71.230	2004	SOUTH	Messenger *et al.,* [12]
92122	92122102R	*Procyon lotor*	TcIV	USA	Georgia			OUTGROUPS	Messenger *et al.,* [12]
CANIII	CANII cl1	*Homo sapiens*	TcIV	Brazil	Belem			OUTGROUPS	Messenger *et al.,* [12]
CM17	CM17	*Dasypus spp.*	TcIII	Colombia	Carimaga			OUTGROUPS	Messenger *et al.,* [12]
X1060	X10610 cl5	*Homo sapiens*	TcIV	Venezuela	Guárico			OUTGROUPS	Messenger *et al.,* [12]
ERA	ERA cl2	*Homo sapiens*	TcIV	Venezuela	Anzoátegui			OUTGROUPS	Messenger *et al.,* [12]
10R26	10R26	*Aotus spp.*	TcIV	Bolivia	Santa Cruz			OUTGROUPS	Messenger *et al.,* [12]
SAIRI3	Saimiri3 cl1	*Saimiri sciureus*	TcIV	Venezuela	Venezuela			OUTGROUPS	Messenger *et al.,* [12]

Isolates from México and Guatemala were characterized to DTU level via the amplification and sequencing of glucose-6-phosphate isomerase (*GPI*) as previously described by Lauthier *et al.,* (2012) [18]. Subsequently, nine maxicircle gene fragments were amplified, sequenced and concatenated from the Méxican and Guatemalan strains according to Messenger *et al*., 2012 (excluding *ND4*) [12]. Phylogenetic analysis was also conducted as in Messenger *et al*., 2012 [12]. Nineteen nuclear microsatellite loci previously described by Llewellyn *et al.,* 2009 [13], were selected based on their level of TcI intra-lineage resolution. Microsatellite loci were amplified across 21 unpublished biological stocks from México and Guatemala. Reaction conditions were as described previously [13]. Dendrograms based on multilocus allele profiles were constructed also according to Llewellyn *et al.,* 2009 [13].

**Figure 1:**
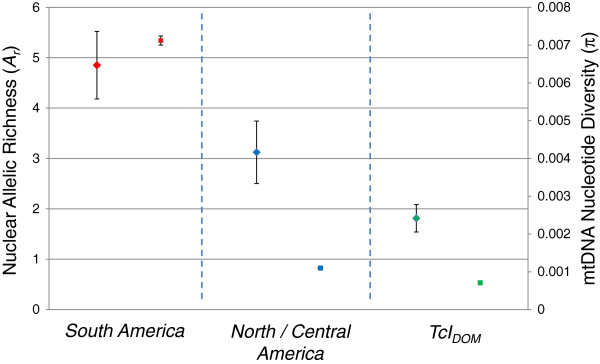
**Nucleotide diversity and allelic richness comparisons across North and South American.***Trypanosoma cruzi* I populations. Left hand data points (diamond) indicate allelic richness ± standard error over loci. Right hand data points (square) indicate nucleotide diversity (π) ± standard error over pair-wise comparisons.

Maxicircle nucleotide diversity (π) was calculated for TcI_NORTH-CENT_, TcI_SOUTH_ and TcI_DOM_ respectively in DNAsp v5 [19]. Nuclear allelic diversity was calculated for the same populations using allelic richness (A_r_) in FSTAT [20]. The resulting values are shown in Figure 1.

Nucleotide sequences per gene fragment are available from GenBank under the accession numbers *MURF1* (fragment a): JX431060 - JX431084; *MURF1* (fragment b): JX431085 - JX431109; *ND1*: JX431110 - JX431134; *ND5* (fragment a): JX431135 - JX431159; *ND5* (fragment b): JX431160 - JX431184; *9S rRNA*: JX431185 - JX431209; *12S rRNA*: JX431210 - JX431234; *COII*: JX431235 - JX431259 and *CYT b*: JX431260 - JX431284.

**Figure 2:**
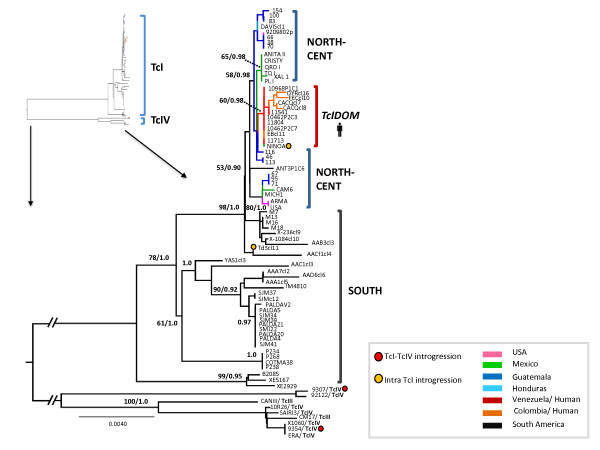
**Isolate grouping of 72 *Trypanosoma cruzi *I strains, as well as outgroups, based on nine concatenated maxicircle sequences.** Bayesian consensus topology is displayed. Bayesian posterior probability analysis (BPP) was performed using MrBAYES v3.1. Five independent analyses were run using a random starting tree with three heated chains and one cold chain over 10 million generations with sampling every 10 simulations (25% burn-in). Decimal values (second number) on nodes indicate Bayesian probabilities for clusters. First number indicates the Maximum-Likelihood (ML) % bootstrap support for clade topologies, which was estimated following the generation of 1000 pseudo-replicate datasets. Branch colours indicate isolate origin. Isolates that show clear incongruity between nuclear genotype and maxicircle genotype are marked. Outgroup branches were cropped for ease of visualization, full branch lengths are show inset top right.

Across the 3,449 bp final concatenated alignment (including outgroups), a total of 374 variable sites were found. The mitochondrial phylogeny supported the presence of significant diversity among the isolates examined (Figure 2). TcI_DOM_ strains formed a monophyletic clade [60% ML BS/0.98 BPP]. The principal division in the phylogeny was between TcI_SOUTH_ and TcI_DOM_/TcI_NORTH-CENT_ (98% ML BS/0.98 BPP). However, this division is incomplete, such that a subset of South American strains is also grouped with TcI_DOM_ and TcI_NORTH-CENT_. Thus, it is not possible to conclude that TcI_DOM_ maxicircle sequences nest uniquely among those from TcI_NORTH-CENT_ strains. Conversely, a basal relationship of the TcI_NORTH-CENT_ to TcI_DOM_ is suggested at the level of nucleotide diversity by population (Figure 1), whereby TcI_DOM_<TcI_NORTH-CENT_<TcI_SOUTH_. Low standard errors about the mean in all three populations, but especially in TcI_DOM_ and TcI_NORTH-CENT_, suggest that sample size had little impact on the accuracy of estimation between populations.

**Figure 3:**
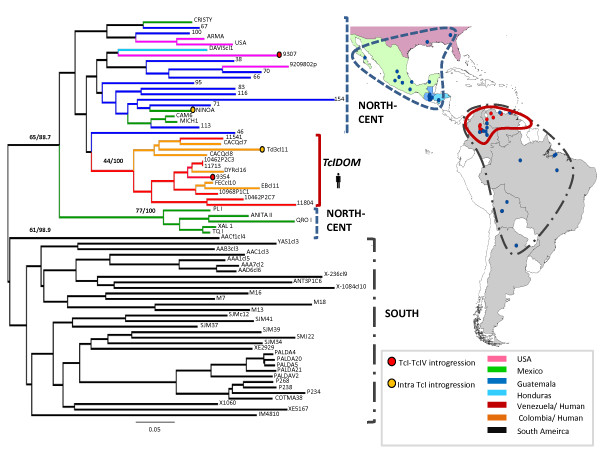
**Isolate grouping of 72 *Trypanosoma cruzi *I strains based on nineteen nuclear micrsoatellite markers.** Neighour-joining clustering algorithm implemented. Bootstrap values are included on important nodes. The first figure indicates % bootstrap support over 10,000 trees, the second the % stability over 1000 trees accounting for multi-allelic loci in the dataset. For further details see Llewellyn *et al*., 2009 [13]. Branch colours indicate isolate origin. The three principal populations TcI_DOM_ TcI_SOUTH_ and TcI_NORTH-CENT_ are shown on both map and tree. Red circles correspond to isolates from TcI_DOM_. Isolates that show clear incongruity between nuclear genotype and maxicircle genotype are marked with reference to Figure 2.

Distance-based clustering using the microsatellite dataset indicated the presence of several well defined clades (Figure 3). Importantly in this case the monophyly of North-Central American isolates was corroborated, and TcI_DOM_ clustered firmly within them (bootstrap 65%). By contrast, South American isolates fall into a divergent but diverse clade. Thus the nuclear data provide stronger support for divergence of TcI_DOM_ from within TcI_NORTH-CENT_ than the maxicircle phylogeny. Sample size-corrected allelic richness estimates are consistent with hierarchical patterns of clustering based on pair-wise genetic distances. As with the maxicircle dataset, there is a pronounced cline in diversity across the populations studied - A_r_ TcI_DOM_< A_r_ TcI_NORTH-CENT_< A_r_ TcI_South_ (Figure 1).

### TcI dispersion into Central and North America

Using a 100 MYA biogeographic calibration point [6], molecular clock analyses point to the origin of *T. cruzi* (*sensu stricto*) 5 – 1 MYA [21-23] and a most recent common ancestor for TcI at 1.3-0.2 MYA [22]. Reduced genetic diversity among North-Central American isolates by comparison to their southern counterparts is powerful evidence in support of others who suggest that TcI originated in South America [13,24]. The emergence of TcI in the South occurred prior to either migration across the Isthmus of Panama alongside didelphid marsupials during the Great American Interchange [25], or perhaps prior to northerly dispersal via volant mammals (e.g. bats).

### Origin of TcI_DOM_

Recent findings indicate a close resemblance between TcI_DOM_ isolates from the northern region of South America and parasite populations from Central and North America by the use of nuclear and mitochondrial markers [11-13]. Indeed SL-IR genotyping suggests a distribution for TcI_DOM_ that now extends as far south as the Argentine Chaco, where multiple sequences have been identified from human and domestic vector sources [14]. Llewellyn *et al*., (2009) originally hypothesised that a distinct human/domestic clade could be maintained despite the presence of nearby infective sylvatic strains due to the low parasite transmission efficiency by the vector [13]. In this case multiple feeds by domestic vector nymphs are required to infect individuals, as such human – human transmission is far more common than reservoir host - human transmission. Originally this hypothesis was developed to explain the epidemiology of Chagas disease in Venezuela. However, TcI_DOM_ is clearly widespread and recent data propose a date for its emergence 23,000 ± 12,000 years ago [11]. This corresponds to the earliest human colonisation of the Americas [26]. Thus it seems that TcI_DOM_ may be as ancient as humans in South America. Crucially, our data, which show that TcI_DOM_ is nested among North and Central American strains, suggest that this widespread domestic *T. cruzi* genotype may actually have made first contact with man in North–Central America.

The expansion of limited diversity genotypes into domestic transmission cycles is a familiar story in *T. cruzi*. This phenomenon seems to have occurred almost simultaneously with TcI_DOM_ (<60,000 YA) in the Southern Cone region but involving DTUs TcV and TcVI [22]. Nonetheless, static human population densities sufficient to support a sustained domestic cycle are presumably vital. For TcI_DOM_, patterns of genetic diversity suggest early colonizing Amerindians may have been responsible for its southerly migration and dispersal from North/Central America. However, such early settler populations were probably small, dynamic, and inherently unsuitable to sustain transmission of such a genotype. Many questions, therefore, remain unanswered regarding its emergence. Insight could perhaps be drawn from a better understanding of the current distribution and diversity of TcI_DOM_ (including samples from the Southern Cone), patterns of vector population migrations, and even from analysis of ancient DNA (e.g. [27]). We hope this report serves to galvanize efforts towards this understanding, especially among researchers in Central and North America, where many of the answers lie.

## Competing interests

The authors declare no competing financial interests. The funder played no role in the study design.

## Authors' contributions

FZE wrote the article, performed the experiments and analysed the data. LAM analysed the data and wrote the article. MAM, TLO, PM, MDL contributed reagents and wrote the paper. CFL analysed the data. JMI, HC, MS contributed reagents. MSL conceived the experiments, analysed the data and wrote the article. All authors read and approved the final version of the manuscript.
